# Root damage induced by intraosseous anesthesia–An in vitro investigation

**DOI:** 10.4317/medoral.18386

**Published:** 2012-12-10

**Authors:** Christian Graetz, Karim M. Fawzy-El-Sayed, Nicole Graetz, Christof-Edmund Dörfer

**Affiliations:** 1 Dr. Med. Dent., Assistant professor. Clinic for Conservative Dentistry and Periodontology, School of Dental Medicine, Christian Albrechts University, Kiel, Germany; 2 Dr. Med. Dent., Assistant professor. Clinic for Conservative Dentistry and Periodontology, School of Dental Medicine Christian Albrechts University, Kiel, Germany. Oral Medicine and Periodontology Department, Faculty of Oral and Dental Medicine, Cairo University; 3 Assistant professor. Clinic for Conservative Dentistry and Periodontology, School of Dental Medicine, Christian Albrechts University, Kiel, Germany; 4 Prof. Dr. Med. Dent., chairman and head of the department. Clinic for Conservative Dentistry and Periodontology, School of Dental Medicine, Christian Albrechts University, Kiel, Germany

## Abstract

Objectives: The principle of the intraosseous anesthesia (IOA) relies on the perforation of the cortical plate of the bone for direct application of the local anesthetic solution into the underlying cancellous structures. During this procedure, IOA needles might accidentally come in contact with the tooth roots. The aim of the current in vitro study was to examine the consequences of this ‘worst case scenario’ comparing five commercially available IOA systems. Material and Methods: Extracted human roots were randomly perforated using five different IOA systems with a drilling time ≤5s. To simulate normal in vivo conditions, the roots were kept humid during the drilling procedure. Data was statistically evaluated using F-test (SPSS16, SPSS Inc., Chicago, USA) and the significance level was set at p≤0.05. Results: All examined systems resulted in root perforation. Drill fractures occurred in either none 0% (Quicksleeper®, Anesto®, Intraflow®, Stabident®) or 100% (X-Tip®) of the applications. Excessive heat generation, as evident by combustion odor as well as metal and tooth discoloration, appeared in 30% (Quicksleeper®), 40% (Anesto®), 60% (Intraflow®), 90% (Stabident®) and 100% (X-Tip®) of all perforations. Conclusion: Within the limits of in-vitro studies, the results show a potential for irreversible root damage that might be inflicted by an improper use of IOA systems.

** Key words:**Intraosseous anesthesia, complication, root damage.

## Introduction

The principle of the intraosseous anesthesia (IOA) relies on the idea of perforating the cortical plate of the bone to apply the local anesthetic solution directly into the underlying cancellous substance (1). Although the technique was initially described almost a century ago (2). Among the proposed advantages of IOA are its reliable profound anesthetic effect and the lack of any annoying numbness of the cheek and lip tissues (1).

Current knowledge on the potential risks of any inferior alveolar nerve block makes it necessary to carefully analyze its risk-benefit ratio and to offer effective alternatives to the patients (3). Among possible complications of the block anesthesia, including an occasional intravascular application or an accidental direct nervous damage, an insufficient anesthetic effect was measured in 40-100% of all applications (4,5). The intraligamentary anesthesia, which is recommended as an ideal alternative (6), leads to an anesthetic success in only 53 -83% of the cases (7), might result in iatrogenic pain and tissue damage and induces bacteremia in almost 100% of the applications (8).

IOA presents a more reliable alternative, although being less popular (9). Literature marks the anesthetic success of IOA to be from 45-100% (10-12). It can achieve an anesthetic effect ranging even from 75-100% (4,11), when employed as an adjunct to classical techniques. The current clinical indication of IOA lies especially in severely painful conditions, as in cases of irreversible pulpitis (13,14). Nevertheless, due to its limited anesthetic duration this method remains only restrictedly suitable for broader surgical interventions (15).

Most of the currently available commercial systems for IOA can be well integrated into everyday practice (16). Possible side effects such as a temporary increase in pulse rate (4,17) are often mentioned in contrast to the rarely reported root damage, drill fractures, postoperative pain or delayed wound healing (18), which represent more serious complications (6). However, there exist no systematic investigations on the side effects of IOA up to now. According to the manufacturer’s instructions, drilling is required to deploy the anesthetic solution between the teeth roots. A possible accidental root damaging effect during IOA application can therefore not be excluded. Additionally, it is difficult to distinguish between IOA perforations of either bone or root substance by tactile means.

In light of this background, the aim of the current in vitro study was to examine and compare the possible root damaging effects of five different IOA systems.

## Material and Methods

-IOA Systems

Five commercially available IOA systems were examined and compared with respect to their possible root damaging effects in vitro ( Table 1). Intraflow® ‘IS’ (Pro-Dex Micro Motors, Santa Ana, USA), Quicksleeper® ‘QS’ (Dental Hi Tec, Cholet Cedex, France) and Anesto® ´AS` (W&H Dentalwerk Bürmoos, Austria) are single-step IOA systems with a rotary drilling syringe, allowing drilling and subsequent anesthetic solution application. In contrast, two-step IOA systems such as Stabident® ‘SD’ (Fairfax Dental Inc, Miami, USA) and X-Tip® ‘XS’ (Dentsply DeTray, Konstanz, Germany) require an additional syringe for applying the anesthetic solution after the initial drilling step. The Quicksleeper® system additionally includes a computer controlled drilling process allowing a digital control of rotation speed, torque and drilling time.

**Table 1:**
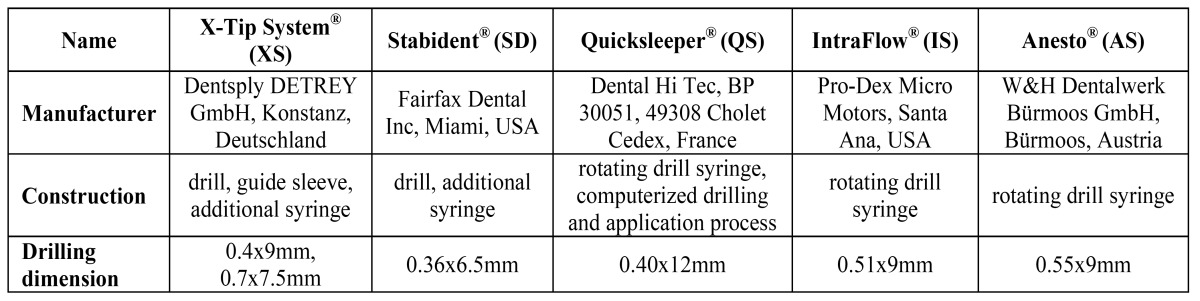
Producer and main details of the tested intraosseous systems.

-Procedure

**Figure 1:**
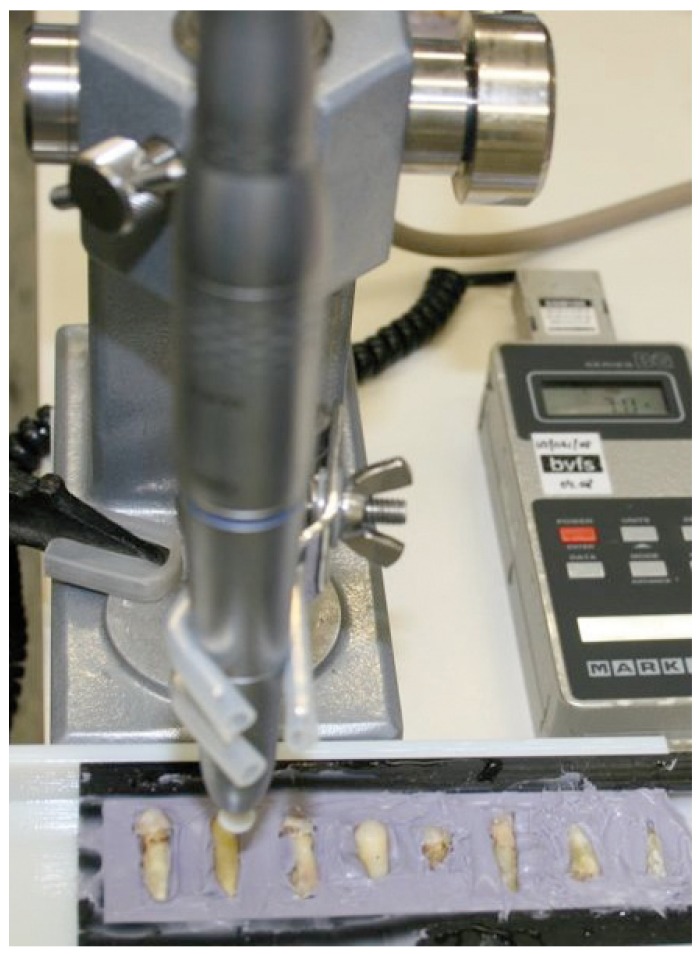
Teeth embedded in a silicone model with a built-in water reservoir. IOA system hand-piece fixed at a 90° angle to the intended line of perforation, using the specially designed splint with a pressure-calibrated support plate.

Ten freshly extracted human teeth (2 cuspids, 2 bicuspids and 6 molars) were immersed for 15 minutes in a 3% NaOCl solution. Following 72 hours of storage in 0.9% NaCl solution, the teeth were kept humid by embedding them in a silicone model with a built-in water reservoir (Flexitime®, heavy, Heraeus, Hanau, Germany) exposing only one third of their facial surfaces inci-so-apically. The hand pieces of the IOA systems under investigation were fixed in a 90° angle to the intended line of perforation, using a specially designed and constructed jig (Fig. 1). The five different IOA systems were tested in random order on each root surface. A total of fifty reproducible drillings (five on each root) were performed on the ten roots. The wet dental hard substances were perforated without the application of any additional external coolant to simulate the intraosseous situation.

-Analysis

**Figure 2:**
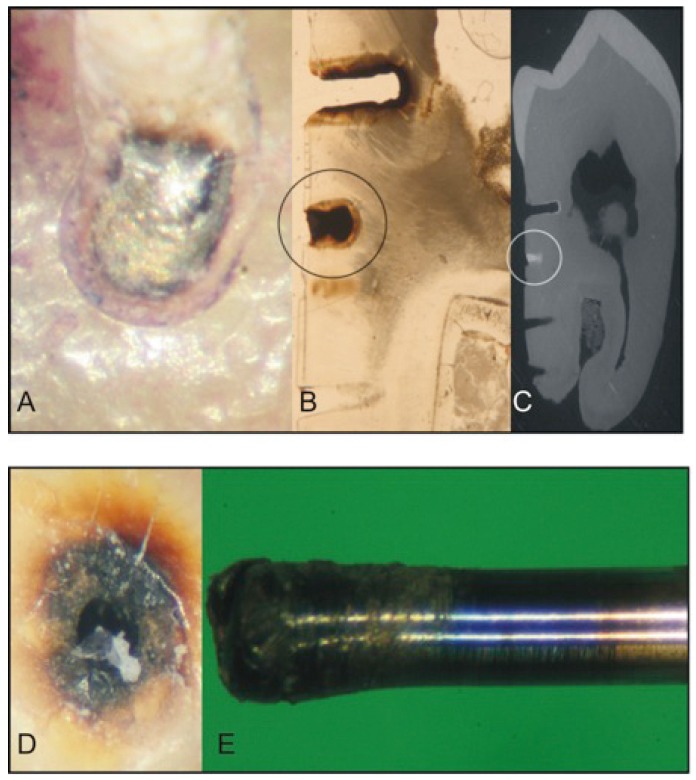
Root surface damage induced after perforation with the X-Tip® System (A-C) and the Stabident® System (D-E). A) In situ appearance (light microscopy, 60 x magnification). B) Oxidation signs (black circle, light microscopy 40 x magnification) and C) fractured fragment (white circle, radiography). D) Color changes of the dental hard substance and E) deformation and color changes of the metal drills as a sign of oxidation due to heat exposure (tempering color) (magnified photography).

The maximum contact force during each drilling process was measured using a pressure-calibrated support plate (Mark 10, Universal Elektronik GmbH, Ronnenberg, Germany). Deformation torque, as measured by the angle of tip rotation on the length axis, attrition of the drills and alteration of the root surfaces were investigated by light microscopy (60x magnification, SZ60, Olympus, Hamburg, Germany) (Fig. 2) and magnified photography. In addition, micro-damages of root substance of four test roots were analyzed (2 bicuspids and 2 molars) by means of microscopy (40x magnification) (Fig. 2) as well as radiography (Fig. 2). Heat generation was noticed by a color change of the dental hard substance (oxidation sign) (Fig. 2) and metal burs (tempering color) (Fig. 2) as well as combustion odor during the drilling procedure.

The Ethical Committee of the Christian-Albrechts-University at Kiel, Germany approved the protocol of the present in-vitro study (AZ: 444/10). Statistical significances were calculated using F-test (SPSS16, SPSS Inc., Chicago, USA) and the significance level was set at p≤0.05.

## Results

-Drilling time and depth

The drilling time for all systems ranged between 4.0s and 5.0s with a mean of 4.5±0.3s ( Table 2). Drilling with the SD, QS and XS system for a specific time resulted in indentations without complete root penetration. IS and AS caused complete root perforation in 70% (IS) and 80% (AS) of all applications respectively. Notably, the tip of the XS system advanced into the root substance until its guiding sleeve touched the tooth surface, preventing the tip from further advancement.

-Deformation and fracture

**Table 2:**
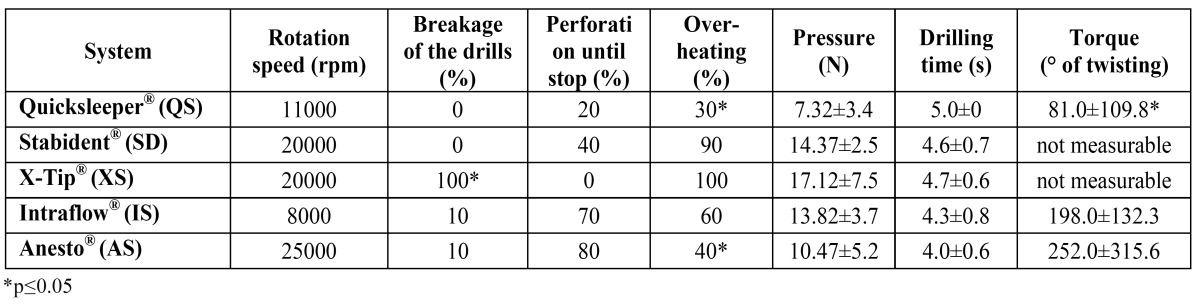
Main results of the five intraosseous systems in percent of all trials.

For the IS and the AS systems, loss of bur material was noted in all applications. On the other hand, drilling with AS, IS and XS systems resulted in drill fractures ( Table 2). The average pressure for the perforation process ranged between 7.3±3.4N (QS) and 17.1±7.5N (XS). A significantly higher incidence of drill breakages (100%) was noted for the XS system.

A significantly lower torque of the advancing needle was further noted for the QS system, lowering its root damaging indentation potential ( Table 2). The torque values for the SD and XS system were not measurable ( Table 2) as the combustion signs (metal tempering color) on the tips of both systems made an angle determination, even under magnification, impossible (Fig. 2).

-Heat induced changes

Heat generation, as evident by combustion odor as well as metal and tooth discoloration, was noted in 30-100% of all applications ( Table 2). For the AS and QS systems significantly less signs of heat generation were noted (30% and 40% respectively).

## Discussion

The initial step of any IOA technique is the perforation of the cortical plate of the bone employing thin drills (full metal drill max. Ø 0.36 mm or drilling syringes max. Ø 0.55 mm). Considering the pressure and speed applied, this would allow cortical bone perforation without the need for an additional coolant (1,19), while inducing no thermal tissue damage (16,20). Therefore, no coolant was incorporated in our experimental design apart from the water reservoir, which kept the roots humid. In systems employing rotating syringes, as the AS, IS and QS systems, the supplied syringe additionally functioned as a drill, whereas in the SD and the XS system an access hole had to be made with a special drill prior to the syringe insertion into the pre-drilled hole.

In the current study, AS and IS systems demonstrated a high cutting efficiency. However, although an associated syringe deformation was noted, the incidence of breakage in these systems remained very low (10% of all applications), indicating a high structural stability and strength of the used syringes in these two systems. A common popular approach to prevent instrument fractures is to control speed, rotation torque and drilling time by computer. This was adopted by the QS system. This computer controlled system allowed even lower syringe diameters (Ø 0.04 mm) to be safely employed without a potential risk of syringe breakage or root perforation as was evident in the QS system showing 0% breakage. Such an event of accidental syringe breakages would harbor great risks as the broken needle would be technically hard to retrieve, especially if such breakages occurred at or below the level of the cortical plate, and could require an extensive surgical procedure to gain access to it.

Compared to systems employing a rotary syringe (AS, IS, QS), the use of full metal drilling systems (SD, XS) significantly increased the risk for thermal damage as was demonstrated by the overheating signs. In all systems under investigation, thermally damaged sites showed typical signs of excessive heating as evident by combustion odor, metal as well as tooth discoloration (21). This short but massive rise in temperature of solid states (50-250°C) could possibly lead to pulpal (22) and periodontal tissue damages (23,24) in vivo. In our in vitro study, the wet hard tooth tissue model (tooth were imbedded in silicon with a reservoir of water) was designed to simulate the root’s thermal conduction in vivo (24). All five tested IOA systems operated according to the manufacturer’s instructions without external coolant usage. Previous in vitro investigations showed intraosseous temperature changes below the tissue damaging threshold for IOA systems (25,26). The higher temperature rise during an accidental intra dental perforation, as noted in the current study, represents an increased risk for thermal tissue damage. The potential thermal damages for the pulp or the periodontium might be similar to those induced by power driven scaling or laser system root surface applications with insufficient cooling (27). An external root resorption (28) or even osteonecrosis with an accompanying bone sequestration might result as a consequence of such an excessive overheating effect (18).

A possible accidental root contact should therefore be absolutely avoided, especially as an earlier in vitro pilot study using an anatomic human model showed no tactile difference between bone or root perforation for IOA systems. Among the proposed preventive strategies are careful clinical examinations to determine any bulges of the cortical plate indicative of underlying roots and preoperative x-rays to determine the location of the roots accurately. In this context the prime importance of profound anatomical knowledge as well as the dentist’s clinical experience have to be underlined. Ultimately, in the event of an accidental root surface contact, all IOA systems investigated in this study might induce an irreversible tooth damaging effect. Irreversible dental injuries can only be avoided with sufficient care and experience of the clinician and a good understanding of the potential risks and restrictions for this anesthetic method.
